# Conjugative plasmids inhibit extracellular electron transfer in *Geobacter sulfurreducens*

**DOI:** 10.3389/fmicb.2023.1150091

**Published:** 2023-03-17

**Authors:** Mathias Fessler, Jonas Stenløkke Madsen, Yifeng Zhang

**Affiliations:** ^1^Department of Environmental and Resource Engineering, Technical University of Denmark, Kongens Lyngby, Denmark; ^2^Section of Microbiology, Department of Biology, University of Copenhagen, Copenhagen, Denmark

**Keywords:** *Geobacter sulfurreducens*, extracellular electron transfer, nanowires, *pilA*, *omcE*, microbial electrochemical systems, conjugative plasmids, pKJK5

## Abstract

*Geobacter sulfurreducens* is part of a specialized group of microbes with the unique ability to exchange electrons with insoluble materials, such as iron oxides and electrodes. Therefore, *G. sulfurreducens* plays an essential role in the biogeochemical iron cycle and microbial electrochemical systems. In *G. sulfurreducens* this ability is primarily dependent on electrically conductive nanowires that link internal electron flow from metabolism to solid electron acceptors in the extracellular environment. Here we show that when carrying conjugative plasmids, which are self-transmissible plasmids that are ubiquitous in environmental bacteria, *G. sulfurreducens* reduces insoluble iron oxides at much slower rates. This was the case for all three conjugative plasmids tested (pKJK5, RP4 and pB10). Growth with electron acceptors that do not require expression of nanowires was, on the other hand, unaffected. Furthermore, iron oxide reduction was also inhibited in *Geobacter chapellei*, but not in *Shewanella oneidensis* where electron export is nanowire-independent. As determined by transcriptomics, presence of pKJK5 reduces transcription of several genes that have been shown to be implicated in extracellular electron transfer in *G. sulfurreducens*, including *pilA* and *omcE*. These results suggest that conjugative plasmids can in fact be very disadvantageous for the bacterial host by imposing specific phenotypic changes, and that these plasmids may contribute to shaping the microbial composition in electrode-respiring biofilms in microbial electrochemical reactors.

## Introduction

Conjugative plasmids exist in virtually all natural environments and are characterized by their ability to spread genes horizontally, which is why they play an important role in prokaryotic evolution (Lerat et al., 2005; Soucy et al., 2015). They often carry advantageous traits, such as resistance to metals and antibiotics (Trevors et al., 1985; Carattoli, 2013), that promote their ecological success in microbial communities. The benefits of plasmid acquisition are dictated by the environmental conditions, and depend on how the plasmid affects the host’s ability to compete with surrounding microbes. Conjugative plasmids are large (often above 60 kb) (Smillie et al., 2010) as they encode numerous genes specific for plasmid replication, maintenance, and transfer, which means they usually come at a metabolic cost for the host (San Millan and Maclean, 2017; San Millan et al., 2018). This cost may lead to deselection for plasmid carriage once the environment changes, however, the fitness cost plasmids impose seems to vary a great deal, as plasmids also persist in the absence of selective pressure (Cottell et al., 2012; Wein et al., 2019). So far, reduction in fitness has been related to the increased metabolic burden of maintaining the large plasmid as well as expression of plasmid-borne genes (San Millan and Maclean, 2017; San Millan et al., 2018), with little focus on the impact of the immediate surroundings. Here we show that in *Geobacter sulfurreducens* conjugative plasmids can interfere with a specific phenotype, nanowire-dependent extracellular electron transfer, while imposing a minimal overall fitness burden when other electron acceptors, that do not require nanowires, are available.

*Geobacter sulfurreducens* is a dissimilatory metal-reducing bacterium involved in the natural metal cycle (Mehta et al., 2005) and a model organism used to study extracellular electron transfer (EET). In contrast to most bacteria, electroactive bacteria such as *G. sulfurreducens* do not rely on soluble electron acceptors to get rid of electrons generated during metabolism. EET permits export of electrons to external electron acceptors such as iron(III) minerals or electrodes, in the absence of soluble alternatives. Despite the on-going discussion of the exact role of PilA, a type IV pilus protein, its importance in electron export in *G. sulfurreducens* is clear (Reguera et al., 2005; Lovley and Walker, 2019; Yalcin and Malvankar, 2020). In the first proposed mechanism for electron export in *G. sulfurreducens*, monomers of the PilA protein serve as the building block for the extracellular part of the pilus itself and form the basis of the electrically conductive pilus/nanowire (Lovley and Walker, 2019). The conductivity itself comes from stacking of the side chains of aromatic amino acids (Vargas et al., 2013; Malvankar et al., 2015). Deletion of the *pilA* gene severely reduces EET ability (Reguera et al., 2005), whilst overexpression has the opposite effect (Yi et al., 2009), underlining the importance of these pili. Recently, however, it has been suggested that PilA is in fact involved in the secretion of nanowires and not the actual electron transfer (Gu et al., 2021). In this model the nanowires are composed of the cytochromes OmcS (Filman et al., 2019; Wang et al., 2019), OmcZ (Yalcin et al., 2020; Wang et al., 2022a), or OmcE (Wang et al., 2022b), which give the wires their conductivity, and the decreased conductivity observed in *pilA* deletion strains is, therefore, attributed to reduced secretion of these cytochromes (Richter et al., 2012; Gu et al., 2021). Regardless of the model, PilA has a central role in EET and in the context of the results presented here, it is not a necessity to know the specific path the electrons take across the membrane.

Due to its efficient EET ability *G. sulfurreducens* has been extensively studied and is commonly enriched in microbial electrochemical systems (MESs) inoculated with environmental samples (Malvankar et al., 2012; Tejedor-Sanz et al., 2018). MESs cover a wide variety of promising technologies, where the unique property of electroactive bacteria is used to clean wastewater and recover energy simultaneously (Wang and Ren, 2013; Palanisamy et al., 2019). Bacteria found in wastewater are rich in conjugative plasmids (Tennstedt et al., 2003), thus, understanding the consequence of plasmid carriage on electrode-respiring bacteria and, ultimately, MESs performance is important.

In nature *Geobacter* species inhabit anaerobic iron(III)-rich environments, including freshwater sediments (Cummings et al., 2000), paddy soils (Hori et al., 2010), and subsurface environments (Holmes et al., 2007), where they participate in microbial dissimilatory iron(III) reduction. Additionally, *Geobacter* species are frequently found in wastewater samples (Malvankar et al., 2012; Tejedor-Sanz et al., 2018). As previously mentioned, conjugative plasmids are also widely distributed and have been isolated from similar environments (Rahube et al., 2014; Nielsen et al., 2015; Dang et al., 2017), and there is evidence of natural encounters between *Geobacter* species and conjugative plasmids, in the form of horizontally acquired DNA (Wagner et al., 2012; Arkhipova et al., 2015; Matassi, 2017). Whilst these DNA uptake events could stem from transformation or transduction, they may well stem from conjugation, considering that *Geobacter* spp. and bacteria carrying conjugative plasmids occupy the same environments and conjugation is an efficient mode of horizontal gene transfer (Nazarian et al., 2018). In support of this, *Geobacter lovleyi* contains a genomic island with a *tra* gene cluster (Wagner et al., 2012), a set of genes encoded on conjugative plasmids needed for plasmid transfer (Smillie et al., 2010).

Despite the prevalent presence of conjugative plasmids across a diverse range of natural environments, knowledge of the effects of external factors on plasmid hosts is limited. Studies have shown that extracellular quorum signals (Zhang and Kerr, 1991) and bacteriophages (Lacey, 1980; Barr et al., 1986) can stimulate plasmid transfer. Additionally, sub-inhibitory concentrations of antibiotics may also promote conjugal transfer of transposable elements (Showsh and Andrews, 1992). Common for these studies is that the influence of extracellular factors on plasmid transfer is the focus. Here, however, we show that the surroundings not only affect the transfer frequency, as we find that several conjugative plasmids inhibit growth of *G. sulfurreducens*, specifically when only solid extracellular electron acceptors are available. To our knowledge, this is the first report of such a drastic and negative effect on a specific host phenotype, underlining that immediate surroundings, such as availability and nature of electron acceptors, are important to consider when assessing plasmid-host interactions. In addition, the results presented here suggest that conjugative plasmids can affect the performance of microbial electrochemical systems.

## Materials and methods

### Bacterial strains and cultivation conditions

The bacterial strains and plasmids used in this study are listed in Table 1. *Escherichia coli* strains were routinely grown in LB medium at 37°C if not otherwise stated. When needed 50 μg/ml of kanamycin or 100 μg/ml streptomycin was added.

**Table 1:** Strains and plasmids used in this study.

Strain or plasmid	Relevant features	Reference or source
Strains		
*Geobacter sulfurreducens* PCA	ATCC no. 51573	Caccavo Jr et al. (1994)
*Geobacter sulfurreducens* Δ*pilA*	*pilA::*Chl^R^	Reguera et al. (2005)
*Geobacter chapellei* 172	DSM no. 13688	Coates et al. (2001)
*Shewanella oneidensis* MR-1	ATCC no. 700550	Venkateswaran et al. (1999)
*Escherichia coli* S17-1	*recA pro hsdR* RP4-2-Tc^R^::Mu-Km^R^::Tn7	Simon et al. (1983)
*Escherichia coli* GeneHogs	Leucine auxotroph	Invitrogen
*Escherichia coli* MG1655*-lacI^q^-mcherry*	Chromosomal *att*Tn7 site blocked	Klümper et al. (2015)
Plasmids		
pKJK5-*att*Tn7	Non-disruptive insertion of *att*Tn7 site	Wang Q. et al. (2022)
pKJK5-*att*Tn7-mcherry*	pKJK5-*att*Tn7::*mcherry*-Km^R^	This study
pKJK5-*att*Tn7-*mcherry* ΔtrbC*	*trbC::*Chl^R^	This study
pKJK5 *traF*::Tn	*traF*::Km^R^	Bahl et al. (2007)
pB10::*gfp*	Str^R^, *gfp*	Van Meervenne et al. (2012)
RP4::*gfp*	Km^R^, *gfp*	Musovic et al. (2014)
RSF1010::*gfp*	Km^R^, P*_A1O4O3_-gfpmut3*	Klümper et al. (2014)
pKD46	Temperature sensitive, expresses λ Red recombinase	Datsenko and Wanner (2000)
pKD3	Source of Chl^R^ for *trbC* deletion	Datsenko and Wanner (2000)
pGRG36-P*_A1O4O3_-mcherry*	Km^R^ and P*_A1O4O3_-mcherry* flanked by Tn7L and Tn7R sequences	Strain collection

*Simply referred to as pKJK5 and pKJK5 Δ*trbC* throughout the article.

*Geobacter sulfurreducens, G. sulfurreducens* Δ*pilA* and *G. chapellei* were cultivated in a minimal medium with 20 mM acetate as electron donor and 50 mM fumarate as electron acceptor at 37°C and 25°C, respectively. The *G. sulfurreducens* Δ*pilA* strain was supplied by Professor Derek Lovley (Reguera et al., 2005). The medium contained the following per liter: 1.5 g NH_4_Cl, 0.6 g Na_2_HPO_4_, 0.1 g KCl, 2.5 g NaHCO_3_, and 10 ml/l trace element solution. The medium was bubbled with a N_2_: CO_2_ (80: 20) gas mixture, adjusted to pH 6.8 and autoclaved. When necessary the medium was supplemented with 200 μg/ml of kanamycin or 400 μg/ml streptomycin. For solid medium 15 g/l agar was added. For the iron(III) reduction assays the fumarate was replaced with 50 mM Fe_2_O_3_ (Sigma-Aldrich, nanopowder, <50 nm particle size) or 50 mM iron(III)-citrate. Anthraquinone-2,6-disulfonate (AQDS) was used at a final concentration of 0.5 mM.

When cultivated aerobically, LB medium was used for *Shewanella oneidensis* MR-1. For anaerobic growth *S. oneidensis* grew with 15 mM lactate and 40 mM fumarate in minimal medium containing the following per liter (Baron et al., 2009): 0.46 g NH_4_Cl, 0.225 g K_2_HPO_4_, 0.225 g of KH_2_PO_4_, 0.117 g MgSO_4_·7H_2_O, 0.225 g (NH_4_)_2_SO_4_, 100 mM HEPES, and 5 ml/l trace element solution. The medium was bubbled with N_2_ gas, adjusted to pH 7.2 and autoclaved. When needed, the medium was supplemented with 50 μg/ml kanamycin. *S. oneidensis* was grown at 25°C.

The trace element solution used for all the above contained per liter: 1.5 g nitrilotriacetic acid, 3.0 g MgSO_4_·7H_2_O, 0.5 g MnSO_4_·H_2_O, 1.0 g NaCl, 0.1 g FeSO_4_·7H_2_O, 0.18 g CoSO_4_·7H_2_O, 0.1 g CaCl_2_·2H_2_O, 0.18 g ZnSO_4_·7H_2_O, 0.01 g CuSO_4_·5·H_2_O, 0.02 g KAl (SO_4_)_2_·12H_2_O, 0.01 g H_3_BO_3_, 0.01 g Na_2_MoO_4_·2H_2_O, 0.03 g NiCl_2_·6H_2_O, 0.3 mg Na_2_SeO_3_·5H_2_O, and 0.4 mg Na_2_WO_4_·2H_2_O.

### Plasmid construction and electroporation

RP4, pB10, pKJK5 *traF*::Tn and RSF1010 were available from our strain collection. For plasmid features see Table 1. pKJK5-*att*Tn7-*mcherry* (simply referred to as pKJK5 throughout the article) was constructed by non-disruptive insertion of *mcherry* and a kanamycin resistance gene from pGRG36-P*_A1O4O3_-mcherry* into pKJK5-*att*Tn7 as previously described (Wang Q. et al., 2022). Once the insertion had been verified with Sanger sequencing, the plasmid was purified with the Plasmid Midi AX kit (A&A Biotechnology) and electroporated into the *E. coli* GeneHogs donor strain. We used this version of pKJK5 instead of the original isolate to ensure we could assess conjugation with flow cytometry if needed.

pKJK5 Δ*trbC* was constructed *via* λ Red recombineering by replacing *trbC* in pKJK5-*att*Tn7-*mcherry* with a chloramphenicol resistance cassette. The chloramphenicol resistance gene was PCR-amplified from pKD3 with primers containing sequences homologous to *trbC* (see Table 2 for primers), and the PCR products were then electroporated into *E. coli* GeneHogs + pKD46 (helper plasmid with ampicillin resistance) and pKJK5. Briefly, the *E. coli* GeneHogs strain with the two plasmids was grown overnight in LB at 30°C, since pKD46 is heat sensitive and does not replicate at 37°C. The next day the culture was diluted 100 fold in LB. After 30 min 100 μl 650 mM arabinose was added to induce expression of genes on pKD46 that facilitate homologous recombination. The culture was grown to OD_600_ = 0.6 followed by incubation on ice for 30 min. Cells were prepared for electroporation by washing and resuspending in 10% glycerol solution. 100 ng PCR product was electroporated into the competent cells with a Bio-Rad Gene Pulser. After incubation for 1 h at 37°C in 1 ml LB, the cells were spread on LB agar plates with 50 μg/ml chloramphenicol and 50 μg/ml kanamycin to select for gene disruption. At 37°C pKD46 cannot replicate, and loss of the vector was verified by plating on LB plates with 100 μg/ml ampicillin. Correct insertion was verified with Sanger sequencing (see Table 2 for primers).

**Table 2:** Primers used in this study.

Primer name	Sequence (5′-3′)	Description
trbC_KO_F	ATGCAAGCACTCTTCCCGTCATTCAGGCTCG ACCAGCGCACATGCAGATTGCAGCATTAC	Knockout of *trbC*. Red seq is complementary to seq in pKJK5, black seq anneals to pKD3 for PCR
trbC_KO_R	TTACCCCGCCACGTAGCCGCGTTCGGCCCAG CGCGTCACCGGAATTAGCCATGGTCCATA	Knockout of *trbC*. Red seq is complementary to seq in pKJK5, black seq anneals to pKD3 for PCR
trbC_seq_F	TAGTCGTTCACATCGCCAG	Seq flanking *trbC*, for sanger sequencing of deletion
trbC_seq_r	CAAGCCCGAGAACATAACC	Seq flanking *trbC*, for sanger sequencing of deletion
pKJK5_tetA_F	TCGTAATTCTGAGCACTGTCG	For verification of pKJK5 conjugation
pKJK5_tetA_R	GCAGGCAGAGCAAGTAGAG	For verification of pKJK5 conjugation

### Filter mating

*E. coli* GeneHogs was used as the plasmid donor for the conjugative plasmids, whilst *E. coli* S17-1 was used for pKJK5 *traF*::Tn, pKJK5 Δ*trbC*, and RSF1010. Conjugations were carried out according to a previously described protocol (Chan et al., 2015). Briefly, 1 ml outgrown aerobic overnight culture of the donor strain was washed twice in LB, then 1 ml growing (OD_600_ around 0.3–0.4) recipient strain was added inside an anaerobic chamber. The cell mixture was centrifuged and the pellet was resuspended in 100 μl residual supernatant and spread on a 0.22 μm filter resting on an agar plate with 0.1% tryptone, inside an anaerobic box. After at least 4 h, the cells were transferred to an agar plate without tryptone to inhibit growth of the donor strain and the appropriate concentration of kanamycin (or streptomycin). This was also done inside the anaerobic box. Once colonies were visible, single colonies were transferred to liquid medium. For pKJK5, successful conjugation was also verified with PCR targeting the *tetA* gene (see Table 2 for primers).

For *S. oneidensis* filter matings were carried out aerobically on LB agar plates followed by selection on M9 agar plates with 15 mM lactate and kanamycin at 25°C.

### Fe(III) oxide and Fe(III)-citrate reduction

Iron(III) oxide assays were performed in 50 ml serum bottles with 25 ml medium. The Fe_2_O_3_ medium was inoculated with 0.5 OD_600_ units of an overnight culture in early stationary phase. Each pair of strains, i.e., the given strain with and without the conjugative plasmid, was inoculated at the same OD_600_ and thus with the same volume, meaning that any potential carryover of small amounts of unused electron acceptor was the same for each pair. For *G. sulfurreducens* 1.35 ml of OD_600_ = 0.37 culture was added, for *G. chapellei* 1.67 ml of OD_600_ = 0.30 culture was added, and for *S. oneidensis* 5 ml of OD_600_ = 0.10 culture was added. After inoculation, the cultures were incubated horizontally on a shaker.

Samples were taken by transferring 400 μl culture to 800 μl 5 M HCl. The iron was dissolved by rotating the samples for 48 h. Samples were then stored at 4°C until all samples had been taken. At this point the Fe^2+^ concentration was measured with ferrozine in 96-well plates by mixing 10 μl sample with 75 μl ferrozine solution (2 g/l ferrozine in 25 mM HCl) and 75 μl acetate buffer (285 g/l sodium acetate in 2 M acetic acid), followed by measuring absorbance at 562 nm. A standard curve was used to convert absorbance to Fe^2+^ concentration.

For the Fe(III)-citrate experiments 400 μl culture was also mixed with 800 μl 5 M HCl, but here the Fe^2+^ concentration was measured immediately.

### RNA sequencing

Cells from growing fumarate cultures were harvested in the exponential phase (at OD_600_ = 0.15) by centrifugation at 12.000 ×*g* for 2 min and 4°C. The pellet was resuspended in Qiagens bacterial RNAprotect reagent, left for 5 min at room temperature before the cells were pelleted and flash frozen and stored at −80°C. Both conditions (i.e., *G. sulfurreducens* with/without pKJK5) were run in triplicates. Cell pellets were sent for RNA extraction and sequencing at Genewiz (Leipzig, Germany). All sequenced samples had a RIN score = 10. The reads were trimmed (Trimmomatic v.0.36), mapped (Star aligner v.2.5.2b) and counted (featureCounts from Subread package v.1.5.2) by Genewiz. Differential gene expression analysis was done with DESeq2. Genes with adjusted *p*-value < 0.05 and log2 fold change below −0.9 or above 0.9 were defined as differentially expressed.

For mapping reads to pKJK5 CLC Genomics Workbench (version 22.0.2) was used.

### Statistical testing

To test if the observed differences in Fe_2_O_3_ reduction were statistically significant unpaired, two-tailed *t*-tests assuming heteroscedasticity were used. The threshold for significance was defined as a *p*-value < 0.05. *t*-tests were performed to test for a difference at the end of the given experiment, i.e., by comparing the last samples of the experiment, except for the growth experiments with fumarate where a difference between doubling times was tested for.

## Results

### pKJK5 specifically inhibits growth on iron oxides in *Geobacter sulfurreducens*

During a preliminary study of *G. sulfurreducens*’ ability to act as plasmid recipient and donor we observed that the conjugative plasmid pKJK5 slowed down growth of *G. sulfurreducens* when growing exclusively with iron oxides as terminal electron acceptors. This immediately caught our attention, as EET is one of the key characteristics of *G. sulfurreducens* responsible for the massive interest in this organism. Until now, EET in *G. sulfurreducens* has been inhibited by *pilA* and cytochrome deletions (Lovley and Walker, 2019; Yalcin and Malvankar, 2020), with the purpose of mapping essential genes for electron export, but natural inhibitors of this defining feature have not been observed previously.

**Figure 1:**
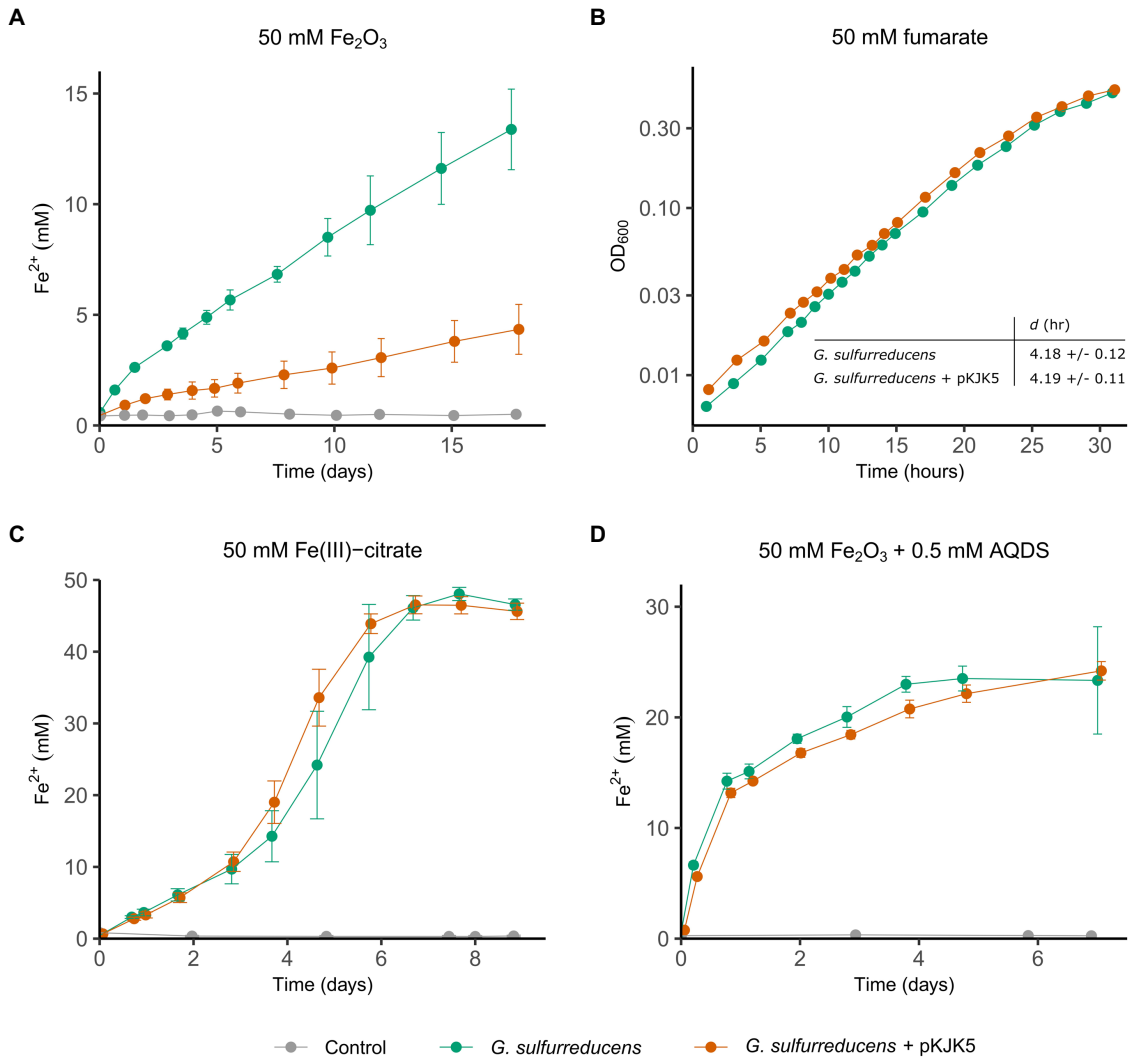
pKJK5 inhibits *G. sulfurreducens*’ ability to reduce Fe_2_O_3_. *G. sulfurreducens* with and without pKJK5 was grown in medium with Fe_2_O_3_ [**(A)**, *n* = 6], fumarate [**(B)**, *n* = 5], Fe(III)-citrate [**(C)**, *n* = 6] or Fe_2_O_3_ + AQDS [**(D)**, *n* = 6] as the only electron acceptor. Growth was either determined by measuring Fe^2+^ concentration **(A,C,D)** or OD_600_ (**B**). For growth on fumarate one representative of five replicates is shown along with doubling times with the standard deviation (SD). All the controls are uninoculated medium. Error bars show SD.

Initially, we assessed and quantified the impact of pKJK5 on the reduction of the iron mineral hematite (Fe_2_O_3_). Hematite is, together with goethite, the most abundant iron oxide in nature (Schwertmann, 1988; Cornell and Schwertmann, 2003; Jiang et al., 2016), which is why we used this as our primary electron acceptor. When it comes to microbial mineral reduction, it is also common practice to prepare more readily reducible iron oxides in the laboratory (Mehta et al., 2005; Reguera et al., 2005; Chan et al., 2015), however, hematite was chosen to better mimic solid electron acceptors encountered in the environment. To assess this, *G. sulfurreducens* was grown in medium with Fe_2_O_3_ as the sole terminal electron acceptor, and under these conditions the conjugative plasmid pKJK5 severely inhibited *G. sulfurreducens*’ ability to reduce iron (Figure 1A). At the end of the experiment, after 17 days, the presence of pKJK5 led to a significant 3-fold decrease in Fe_2_O_3_ reduction (*p* < 0.05). The observed difference could principally be due to the increased metabolic burden of maintaining pKJK5. To clarify whether this was the case, growth of *G. sulfurreducens* on two soluble electron acceptors, fumarate and Fe(III)-citrate, was assessed (Figures 1B,C). Fumarate reduction takes place in the cytoplasm (Galushko and Schink, 2000), whilst Fe(III)-citrate is reduced extracellularly by cytochromes located in the outer membrane (Liu et al., 2015). Growth on these electron acceptors was not affected by pKJK5 (fumarate doubling time: *p* > 0.05, Fe(III)-citrate day 9: *p* > 0.05), suggesting that the plasmid interferes with the specific electron transfer mechanism for reduction of Fe_2_O_3_ rather than imposing a general fitness reduction. In accordance with this, the negative effect of pKJK5 on Fe_2_O_3_ reduction was alleviated by adding the electron shuttle anthraquinone-2,6-disulfonate (AQDS) (Figure 1D) (*p* > 0.05, day 7). For reduction of AQDS *G. sulfurreducens* relies on several outer surface c-type cytochromes (Voordeckers et al., 2010), rather than conductive nanowires, which allowed *G. sulfurreducens* to circumvent the nanowire-dependent electron transfer pathway otherwise needed for growth on iron oxides (Reguera et al., 2005).

### pKJK5 only interferes with extracellular electron transfer mediated by nanowires

**Figure 2:**
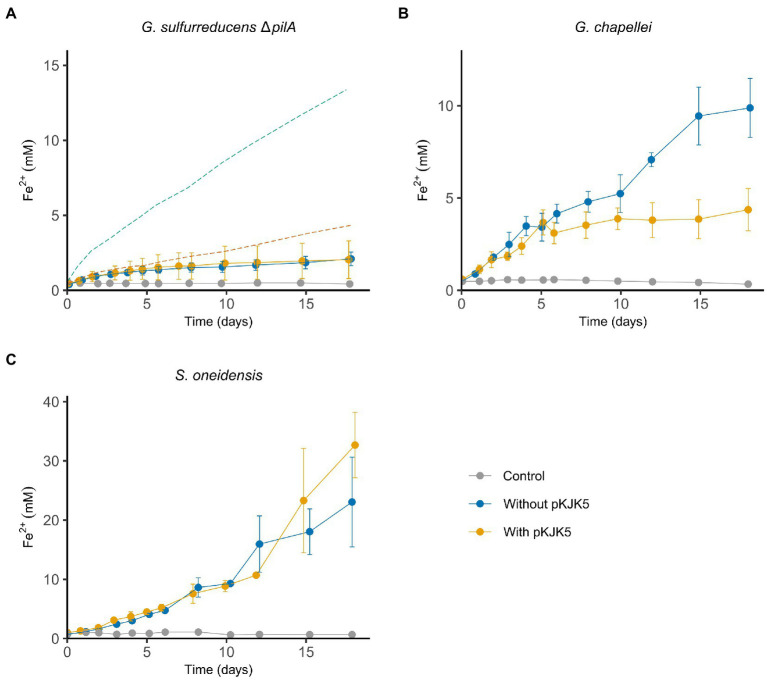
pKJK5 specifically inhibits *pilA*-dependent iron oxide reduction. Reduction of Fe_2_O_3_ by *G. sulfurreducens* Δ*pilA* [**(A)**, *n* = 6], *G. chapellei* [**(B)**, *n* = 3] and *S. oneidensis* [**(C)**, *n* = 3]. Stippled lines **(A)** show Fe_2_O_3_ reduction of *G. sulfurreducens* WT (green) and *G. sulfurreducens* WT + pKJK5 (orange) from Figure 1A to ease comparison. All the controls are uninoculated medium. Error bars show SD.

By now, the importance of the *pilA* gene for extracellular electron transfer to minerals and electrodes in *G. sulfurreducens* is well established (Reguera et al., 2005; Yi et al., 2009), despite some uncertainty on the specific mechanistic role of PilA (Lovley and Walker, 2019; Yalcin and Malvankar, 2020). When expressing pili with low conductivity, electron export to iron oxides and electrodes decreases radically (Vargas et al., 2013; Xing et al., 2014). In addition, *pilA* deletion mutants fail to accumulate OmcZ in the extracellular matrix in biofilms, which also reduces *G. sulfurreducens*’ ability to generate current (Steidl et al., 2016). Whether PilA is involved in electron transport, secretion of cytochromes, or both, the PilA protein is central to both the proposed EET models and clearly essential for EET in *G. sulfurreducens*. This means that growth on insoluble electron acceptors is primarily restricted to PilA-dependent EET pathway(s). In other words, *pilA* is one of the main differentiators between respiration on Fe_2_O_3_ and respiration on fumarate, Fe(III)-citrate, and AQDS. For this reason, our attention turned to this gene. Since our initial experiments indicated that pKJK5 interfered with the microbial nanowires, we conjugated pKJK5 into a *G. sulfurreducens* strain where *pilA* had been deleted. Fe_2_O_3_ reduction was similar in the Δ*pilA* strain with and without pKJK5 (Figure 2A) (*p* > 0.05, day 17), which is consistent with the initial observation and indicates that pKJK5 affects PilA-dependent electron export. In agreement with previous reports, *G. sulfurreducens*’ ability to transfer electrons to iron minerals was reduced but not completely lost in the *pilA* deletion strain (Smith et al., 2014; Ueki et al., 2019).

*G. sulfurreducens* is the most well studied species in the *Geobacter* genus, but other *Geobacter* species also show electroactive properties (Rotaru et al., 2015) and expression of nanowires (Tremblay et al., 2012; Tan et al., 2016). To determine if pKJK5’s effect was common for the *Geobacter* genus or specific for *G. sulfurreducens*, iron oxide reduction by *Geobacter chapellei* was assessed. After preliminary experiments including *Geobacter chapellei*, *Geobacter metallireducens*, *Geobacter bremensis*, and *Geobacter bemidjensis*, it was decided to focus on *G. chapellei* as it was both easy to cultivate and displayed proficient growth on Fe_2_O_3_. Also, *G. chapellei* is likely to use nanowires for EET based on sequence homology (NCBI protein ID = WP_214296113.1). The putative *pilA* gene in *G. chapellei* shows 79% similarity on DNA level and 88% similarity at amino acid level to the *pilA* gene of *G. sulfurreducens* (Supplementary Figure S1). In addition, all five aromatic amino acids that are essential for the conductivity of the pili are conserved in *G. chapellei* (Malvankar et al., 2015). pKJK5 was conjugated into *G. chapellei* and had a similar effect on iron oxide reduction as in *G. sulfurreducens* (Figure 2B). The lowered iron reduction was also statistically significant in *G. chapellei* (*p* < 0.05, day 18). Knowing that pKJK5 did not affect growth on fumarate, ferric citrate or AQDS (Figures 1B–D) this strongly suggests that pKJK5 specifically interferes with *G. sulfurreducens’* nanowires. Further evidence for this was found in the fact that pKJK5 did not inhibit mineral reduction in *Shewanella oneidensis* (*p* > 0.05, day 18), that does not use PilA-dependent nanowires to reduce external electron acceptors (Figure 2C). In *S. oneidensis*, MtrC, a c-type cytochrome anchored in the outer membrane, is the final protein in the electron export pathway (Coursolle and Gralnick, 2012).

### Inhibition of extracellular electron transfer is a general feature of conjugative plasmids

**Figure 3:**
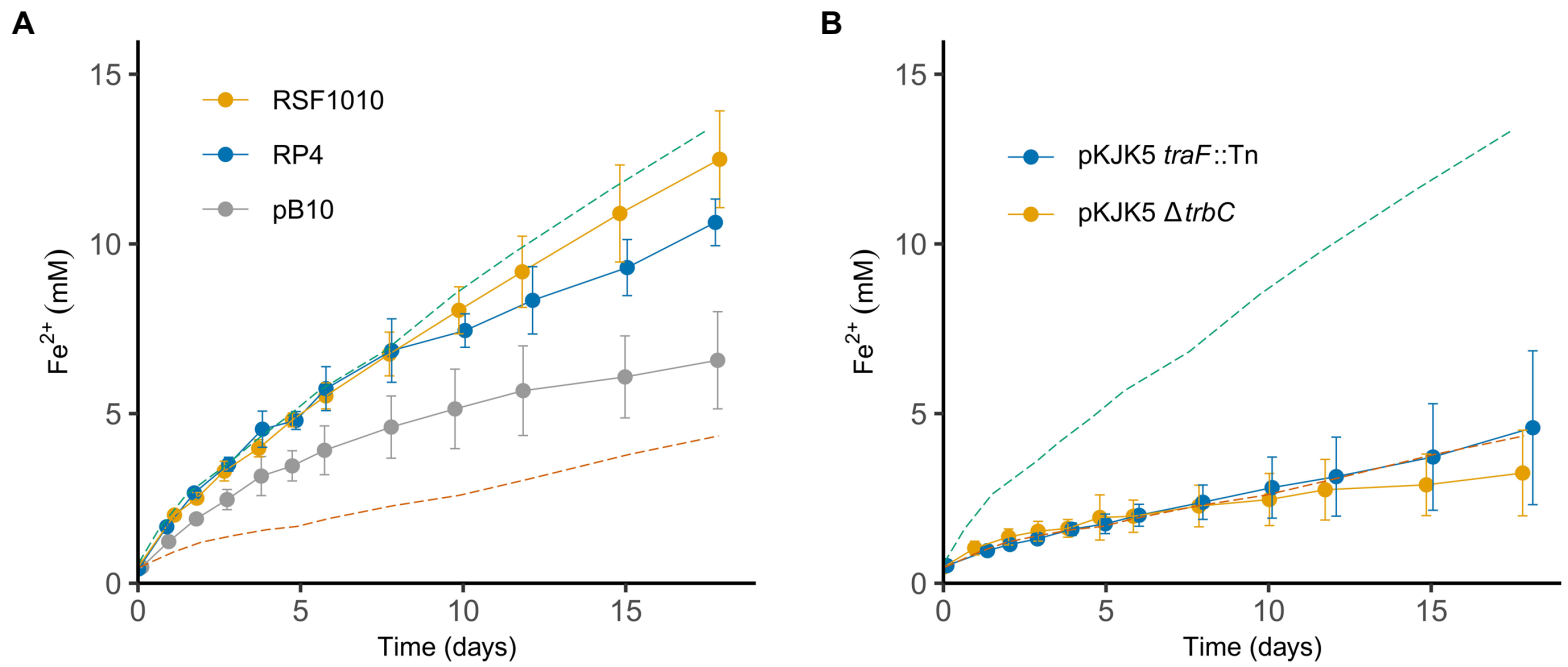
Inhibition of iron reduction in *G. sulfurreducens* is a general feature of conjugative plasmids, but does not depend on the conjugative pilus. Fe_2_O_3_ reduction by *G. sulfurreducens* with three different plasmids [**(A)**, *n* = 6]; pB10 (conjugative), RP4 (conjugative) and RSF1010 (mobilzable), and with two non-conjugative versions of pKJK5 [**(B)**, *n* = 6]. Stippled lines show Fe_2_O_3_ reduction of *G. sulfurreducens* WT (green) and *G. sulfurreducens* WT + pKJK5 (orange) from Figure 1A to ease comparison. Error bars show SD.

pKJK5 is just one of many conjugative plasmids found in nature and, therefore, it is important to establish if the observed phenotype in the two *Geobacter* species is restricted to pKJK5 or if this is a more general feature of conjugative plasmids. To do so we used three additional wild type plasmids: RP4, pB10 and RSF1010(Datta et al., 1971; Scholz et al., 1989; Schlüter et al., 2003). The former two are conjugative plasmids belonging to the incP group like pKJK5, whilst RSF1010 is mobilizable rather than conjugative and belongs to the incQ group. Mobilizable plasmids can transfer upon cell–cell contact just as conjugative plasmids, however, as opposed to conjugative plasmids they do not encoded all the genes needed for this process themselves (Smillie et al., 2010). As seen in Figure 3A the inhibitory effect of conjugative plasmids was not only limited to pKJK5. Even though pKJK5 had the most substantial impact of the plasmids tested, similar patterns were observed for RP4 and pB10 and both plasmids led to a statistically significant decrease in Fe_2_O_3_ reduction (*p* < 0.05, for both plasmids on day 17). On the other hand, the growth of *G. sulfurreducens* on Fe_2_O_3_ was not significantly affected by the mobilizable plasmid RSF1010 (Figure 3A) (*p* > 0.05, day 17). For conjugation four elements encoded on the conjugative plasmid itself are key: an origin of transfer (oriT), relaxases that initiate the DNA transfer at the oriT, type 4 coupling proteins (T4CP), and a type 4 secretion system (T4SS), through which the DNA is transferred (Smillie et al., 2010). As opposed to conjugative plasmids, mobilizable plasmids do not encode a pilus but only the oriT and relaxase (and in some cases the T4CP), why they are not self-transmissible. Therefore, as only the conjugative plasmids had an impact on the Fe_2_O_3_ reduction, these findings suggest that the T4SS (which includes the conjugative pilus) could be responsible for the observed phenotype.

As the data presented so far indicated that the plasmid-mediated inhibition was specific for the nanowire electron transport pathway, the mechanism behind this became our focus. Even though the core pilin proteins are different, both the conjugative pilus and the PilA pilus in *G. sulfurreducens* belong to the family of type IV pili (Giltner et al., 2012). In addition, the conjugative pilus is one of the main differentiators between conjugative and mobilizable plasmids and, therefore, we investigated if the conjugative pilus physically interfered with PilA-mediated EET. To test this, two versions of pKJK5 were used – one with a knock out in *traF*, a gene encoding a protein involved in pilus maturation, and another with a deletion of *trbC*, the gene encoding the conjugative pilus building block (Haase and Lanka, 1997). Both of these pKJK5 versions were non-conjugative (data not shown), but neither of the two alleviated the effect of pKJK5 (Figure 3B) (*p* > 0.05, for both plasmids at the end of the experiment), suggesting that the inhibition is not mediated by the actual conjugative pili. However, there are several other genes involved in biogenesis of the conjugative pilus (Firth et al., 1996), why the finding that neither the TraF nor the TrbC protein alone is responsible for the phenotype is not sufficient to dismiss the conjugative T4SS.

**Figure 4:**
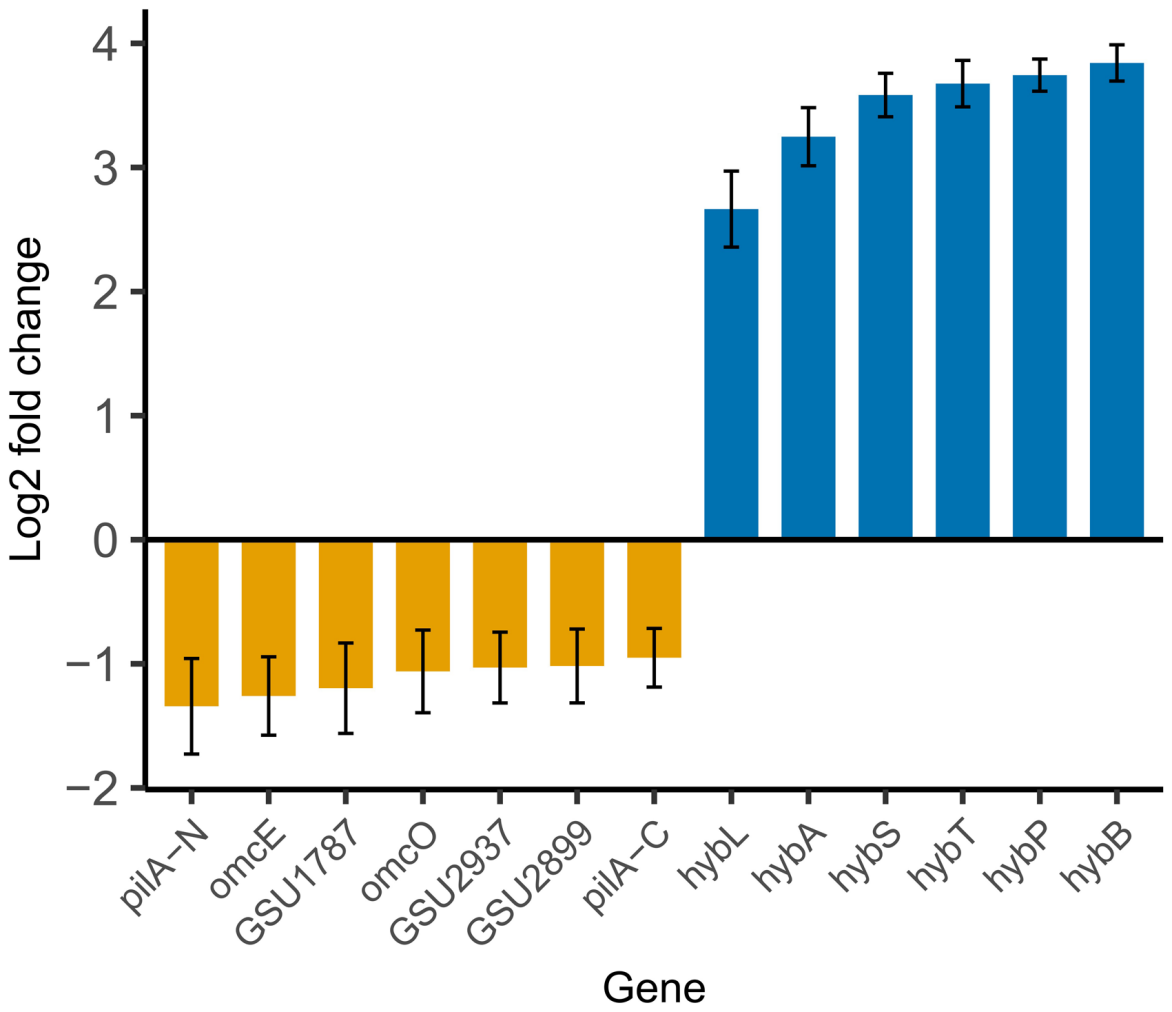
pKJK5 downregulates transcription of *pilA* and several cytochromes in *G. sulfurreducens*. Gene transcription of *G. sulfurreducens* with pKJK5 compared to gene transcription of *G. sulfurreducens* without pKJK5. GSU1787, GSU2937 and GSU2899 encode c-type cytochromes. Error bars show SD (*n* = 3). For full table of differentially transcribed genes see Supplementary Table S2.

Next, we looked into effects of pKJK5 on the host transcriptome, to determine if plasmid-borne genes interfered with expression of genes needed for EET. *G*. *sulfurreducens* is resistant to kanamycin, when harboring pKJK5, and is able to function as plasmid donor (data not shown), which confirms that plasmid encoded genes were expressed in *G. sulfurreducens*. In addition, the transcriptomic data presented below confirmed that pKJK5 genes were transcribed (Supplementary Table S1). pKJK5 led to differential transcription of 81 genes, after removing genes annotated as either hypothetical proteins with unknown function or pseudogenes (Supplementary Table S2). 64 genes were transcribed at reduced levels and 17 genes were induced. The majority of differentially transcribed genes are part of basic cell metabolism, such as replication, transcription, translation and biosynthesis (see Supplementary Table S2 for full list); all processes that are also involved in maintenance of the plasmid. This is in agreement with previous findings (Rösch et al., 2014). In the context of extracellular electron transfer, the analysis showed reduced transcription of both *pilA-N* and *pilA-C* as well as five c-type cytochrome genes (Figure 4). PilA-N (also referred to simply as PilA throughout the article) is the protein that constitutes the nanowire and/or is responsible for cytochrome secretion. PilA-C is, on the other hand, non-conductive (Liu et al., 2020) and also part of the cytochrome secretion complex (Gu et al., 2021). Evidence suggest they were once a single gene (Liu et al., 2020). When *G. sulfurreducens* contained pKJK5, the transcription of *pilA* was reduced with 60% compared to transcription in the plasmid-free cells (adjusted *p-*value <0.05), and the cytochromes were reduced with 58 to 51% (adjusted *p-*value <0.05, for all cytochromes). This strongly implies why *G. sulfurreducens*’ ability to reduce iron minerals diminishes in the presence of pKJK5. Two of the five downregulated cytochromes (OmcE and OmcO) are located in the outer membrane, cytochrome GSU2937 is predicted to localize in the periplasm (Aklujkar et al., 2013), whilst the cellular location of the remaining two unnamed cytochromes is unknown. When *omcE* is deleted the ability of *G. sulfurreducens* to reduce iron oxides is limited (Mehta et al., 2005), and recently OmcE was in fact found to assemble into conductive filaments (Wang et al., 2022b), similar to OmcS and OmcZ filaments (Filman et al., 2019; Wang et al., 2022a). Of the 17 genes that were induced, six genes in particular were highly upregulated. Based on sequence homology all of these, except for *hybT*, encode proteins of a periplasmic membrane-bound [NiFe]-hydrogenase, an enzyme that catalyzes reversible conversion of H_2_ to protons and electrons.

## Discussion

The results presented here demonstrate that pKJK5 inhibits *G. sulfurreducens*’ growth on Fe_2_O_3_ and that this is due to reduced transcription of *pilA* and several c-type cytochromes. However, what causes this reduced transcription is not clear from the RNA sequencing. Considering that PilA and the conjugative pilus are both type IV pili, we speculate that regulation of *pilA* transcription is recognized by elements regulating transcription of the conjugative pilus, which would explain the lower transcription of both *pilA-N* and *pilA-C* (Figure 4). Meanwhile, the reduced transcription of c-type cytochromes is more surprising. Previous reports show upregulation of OmcE, OmcO and GSU2937 in response to iron oxide-dependent growth (Aklujkar et al., 2013), but it is not clear how or why pKJK5 affects the transcription of these genes. At this time, it is best explained as an indirect effect of pKJK5, in the sense that these cytochromes are somehow indirectly coupled to *pilA* expression. Considering that PilA is needed for secretion of OmcS and OmcZ (Gu et al., 2021), this might also be the case for some of the cytochromes that are downregulated in our differential transcription analysis (Figure 4), such as OmcE, which is known to form nanowires (Wang et al., 2022b). If PilA is responsible for secretion of these cytochromes it seems plausible that their expression is coupled to the expression of *pilA*, in order to prevent wasting resources on synthesis of cytochromes in situations where they cannot translocate to the outside of the cell. However, with such cross-regulation *omcS* and *omcZ* would also be expected to show up in the gene expression analysis as these depend on *pilA* for secretion (Gu et al., 2021). Downregulation of these two cytochrome genes was observed, but was not statistically significant (Supplementary Table S2). Interestingly, whilst deletion of *omcE* in *G. sulfurreducens* has no effect on conductivity when respiring on electrodes (Malvankar et al., 2012), iron oxide reduction is slower without OmcE (Mehta et al., 2005). Therefore, the reduced transcription of *omcE* we observe here may also contribute to the poor reduction of Fe_2_O_3_. OmcO, on the other hand, is not essential for iron oxide reduction (Aklujkar et al., 2013), and the remaining three cytochromes have not yet been examined. Since hematite reduction was inhibited by all three conjugative plasmids tested, but not the mobilizable plasmid RSF1010 (Figure 3A), this suggests that the inhibition is caused directly or indirectly by one or more factors encoded as part of the IncP-1 backbone which is similar between pKJK5, pB10 and RP4. Further investigations are needed for identification of the exact mechanism.

As for the increased transcription of the *hyb* genes, we also consider this an indirect effect. The *hyb* genes encode a periplasmic [NiFe]-hydrogenase and we suspect that these genes are also linked to *pilA* and/or cytochrome expression, simply because this seems more plausible than pKJK5 directly regulating *hyb* expression. In *G. sulfurreducens* the *hyb* operon couples hydrogen oxidation to reduction of both soluble and insoluble electron acceptors (Coppi et al., 2004), and upregulation of [NiFe]-hydrogenases is linked to growth on iron minerals (Aklujkar et al., 2013). Here, the observed *hyb* upregulation might be a response to the pKJK5-mediated nanowire downregulation, as these hydrogenases present an alternative route for electron disposal, i.e., by conversion of electrons and protons to H_2_. We want to note that to obtain sufficient biomass for RNA sequencing, the RNA was purified from cultures grown with fumarate and not hematite. We believe this to be an acceptable compromise as the results of the transcription analysis fit well with the phenotypes observed when *G. sulfurreducens* grew with Fe_2_O_3_. This also suggests that the transcription of pKJK5 genes was not affected by the type of electron acceptor.

Often acquisition of conjugative plasmids is associated with a benefit for the bacterial host, such as resistance to antibiotics or heavy metals. However, here we report the opposite, conjugative plasmids severely limit the growth of *G. sulfurreducens* and *G. chapellei*, specifically when respiring on insoluble electron acceptors. Granted, this negative effect is highly dependent on the surrounding environment, however, the inhibition is specific to the very environment *Geobacter* species have specialized to inhabit. Our results suggest that when a plasmid protects against an environmental stressor, there is both a selection and counter-selection for plasmid uptake by *Geobacter* spp., given that the availability of soluble electron acceptors is scarce. In sediments, this means that the availability of electron acceptors may, in fact, be an indirect determinant of conjugal transfer efficiency by preventing proliferation of nanowire-dependent plasmid recipients.

In addition to their potential influence in natural environments, conjugative plasmids may also have an impact on the community structure in artificial systems, namely in microbial electrochemical systems. The configuration and purpose of MESs is very diverse, but common for all these systems are that electroactive bacteria are essential (Wang and Ren, 2013). Whether they respire on the anode, cathode, or both, electron flow between the chambers is an integral part of the reactors. For this reason, the selective pressure for electroactive species is strong and, therefore, it is usually sufficient to inoculate with a diverse mixture of bacteria. Ultimately, electroactive species will dominate the electrode biofilm, why wastewater samples are often used as the inoculum due to their high bacterial diversity (Palanisamy et al., 2019; Wu et al., 2019). Additionally, to achieve sustainable operation, most reactors are designed to run using wastewater as a source of organics. Consequently, there is a continuous entry point for conjugative plasmids, as these are abundant in wastewater (Dröge et al., 2000; Tennstedt et al., 2003; Moura et al., 2012).

As we have shown here, conjugative plasmids repress the transcription of *pilA* and numerous cytochromes, why it is certainly plausible that such plasmids influence the microbial composition in MESs. For *Geobacter* species, commonly enriched in MESs (Kiely et al., 2011; Malvankar et al., 2012; Pepè Sciarria et al., 2019), our results suggest there is a trade-off between the ability to grow on electrodes and the potential positive attributes plasmids can provide, such as the ability to withstand the residual amounts of antibiotics that are found in wastewater (Hirsch et al., 1999; Kortesmäki et al., 2020). In support of this, wastewaters with higher concentrations of antibiotics show increased abundance of antibiotic resistance genes (Rowe et al., 2017). Additionally, conjugative plasmids are implicated in biofilm formation and stabilization (Madsen et al., 2012), underlining their usefulness for the bacterial communities, which complicates the situation even further. In the context of MES community composition, our results also indicate that spread of conjugative plasmids in MESs favor growth of electroactive bacteria that do not rely on nanowires, such as *Shewanella* species. Having said this, biofilms are very complex. Different species fill different roles in biofilms and, therefore, all members of the biofilm do not necessarily need the plasmid even if the surroundings contain residual amounts of antibiotics. In electrode-respiring biofilms, *Geobacter* is more abundant in the inner layers than in the outer layers (Malvankar et al., 2012). This means that toxic or anti-bacterial compounds might never reach the inner biofilm, as they may be removed by plasmid-containing cells in the outer layer. Effectively this gives *Geobacter* species protection without compromising EET ability.

At this point, it is important to note that we are not claiming that conjugative plasmids are a major determinant of microbial community structure in MESs. We argue that they may play a part and that environmental factors are important to consider in regard to MESs community dynamics; thriving in these systems is not simply a question of whether an organism is electroactive or not.

Having established that an important group (IncP) of conjugative plasmids inhibits extracellular electron transfer in pure cultures of *G. sulfurreducens* and *G. chapellei* it is important to better mimic conditions encountered both in nature and MESs, moving forward. This will make it possible to assess the significance of conjugative plasmids in multispecies electroactive biofilms and, thus, better understand how and if they influence MES performance.

## Limitations of the study

For the RNA sequencing it was not possible to obtain sufficient biomass from Fe_2_O_3_ cell cultures, instead RNA was purified from fumarate cultures. RNA was only purified from *G. sulfurreducens* containing pKJK5, not from *G. sulfurreducens* containing the remaining plasmids.

## Data availability statement

The data presented in the study are deposited in the NCBI repository, accession number PRJNA890616.

## Author contributions

MF, JM, and YZ conceptualized the project and wrote the manuscript. MF performed experiments. YZ secured funding. All authors contributed to the article and approved the submitted version.

## Funding

This work was financially supported by the Carlsberg Foundation Distinguished Fellowships (CF18-0084, Denmark).

## Conflict of interest

The authors declare that the research was conducted in the absence of any commercial or financial relationships that could be construed as a potential conflict of interest.

## Publisher’s note

All claims expressed in this article are solely those of the authors and do not necessarily represent those of their affiliated organizations, or those of the publisher, the editors and the reviewers. Any product that may be evaluated in this article, or claim that may be made by its manufacturer, is not guaranteed or endorsed by the publisher.
