# Supplementary material for: Conjugative plasmids inhibit extracellular electron transfer in Geobacter sulfurreducens
Source: Front Microbiol. 2023 Mar 17;14:1150091. doi: 10.3389/fmicb.2023.1150091 (PMC10063792; doi:10.3389/fmicb.2023.1150091)
Supplement: Supplementary file 1 [file Data_Sheet_1.PDF]

A

```
G.chapellei        AGGAGCACAACCAAGGAAAGGAGAAACACATGTTACAGAA 40
G.sulfurreducens   AGCAGCAAAAGAA-GAAAGGAGACACTTATGCTTCAGAA 39

G.chapellei        AATGAGAAACAGAAAAGGTTTTACCCTGATCGAGCTGCTG 80
G.sulfurreducens   ACTCAGAAACAGGAAAGGTTTCACCCTTATCGAGCTGCTG 79

G.chapellei        ATCGTTGTTGCGATCATCGGTATCCTGGCTGCCGTTGCCA 120
G.sulfurreducens   ATCGTCGTTGCGATCATCGGTATTCTCGCTGCAATTGCGA 119

G.chapellei        TCCCGCAGTTTTCATCCTATCGCGTTAAGGCTTACAACAG 160
G.sulfurreducens   TTCCGCAGTTCTCGGCGTATCGTGTCAAGGCGTACAACAG 159

G.chapellei        TGCTGCATCCAGTGATTTGAGGAACTTGAAAACAGGTTTA 200
G.sulfurreducens   CGCGGCGTCAAGCGACTTGAGAAACCTGAAGACTGCTCTT 199

G.chapellei        GAGGCCGCTTTTTCTGAT 218
G.sulfurreducens   GAGTCCGCATTTGCTGAT 217
```

B

```
G.chapellei        MLQKMRNRKGFTLIELLIVVAIIGILAAVAIPQFSSYRVK 40
G.sulfurreducens   MLQKLRNRKGFTLIELLIVVAIIGILAAIAIPQFSAYRVK 40

G.chapellei        AYNSAASSDLRNLKTGLEAAFSDNQYYP 68
G.sulfurreducens   AYNSAASSDLRNLKTALESAFADDQTYP 68
```

Supplementary Figure 1. DNA (**A**) and amino acid (**B**) alignment of presumed e-pili gene in *G. chapellei* (WP_214296113.1) and *G. sulfurreducens pilA* gene (GSU1496). The amino acids marked in yellow are the five aromatic amino acids that are essential for conductivity. The alignments were made with NCBI blast, and the coloring was done with the Color Align Conservation online tool (https://www.bioinformatics.org/sms2/color_align_cons.html).
